# Short-Term Effects of PJE Administration on Metabolic Parameters in Diet-Induced Obesity Mice

**DOI:** 10.3390/foods12081675

**Published:** 2023-04-17

**Authors:** Jun-Hui Choi, Ki-Man Kim, Se-Eun Park, Myung-Kon Kim, Seung Kim

**Affiliations:** 1 Department of Health Functional Food, Gwangju University, Gwangju 61743, Republic of Korea; sekai0572@naver.com (J.-H.C.); kmkim@gwangju.ac.kr (K.-M.K.);; 2 Department of Food Science and Technology, Chonbuk National University, Iksan 54596, Republic of Korea

**Keywords:** *Petasites japonicus* (Siebold & Zucc.) Maxim., diet-induced obesity, metabolic parameter, fat accumulation, anti-obesity

## Abstract

The study investigated the effects of *Petasites japonicus* (Siebold & Zucc.) Maxim. extract (PJE) and fenofibrate on diet-induced obesity (DIO) in mice. PJE was found to contain various bio-active polyphenolic compounds, including kaempferol, p-hydroxybenzoic acid, ferulic acid, gallic acid, chlorogenic acid, 3,4-dicaffeoylquinic acid, caffeic acid, quercetin, rutin, protocatechuic acid, 3,5-dicaffeoylquinic acid, 4,5-dicaffeoylquinic acid, p-coumaric acid, apigenin, and 1,3-dicaffeoylquinic acid. The results showed that PJE treatment up to 1000 μg/mL did not affect the viability of 3T3-L1 cell line, and it reduced the feed efficiency ratio in DIO mice. PJE administration also resulted in a significant reduction in body weight gain and fat accumulation in the liver compared to the DIO control group. Additionally, PJE administration improved the levels of lipid and related parameters, including total cholesterol, triacylglycerol, low-density lipoprotein, very low-density lipoprotein, glucose, insulin, insulin resistance, leptin, and atherogenic or cardiac indexes compared to the DIO control group. The study suggested that PJE may have a beneficial effect on insulin resistance, lipid profiles, atherogenesis, adipokines, and cardiac risk associated with diet-induced obesity.

## 1. Introduction

In recent decades, the prevalence of overweight and obesity has steadily increased due to changes in diet and lifestyle. Of the world’s estimated 650 million adults, 13% were obese in 2016, and by 2030, it is estimated that 19.7% of the population of the world will be obese [1]. In the case of Korea, the adult obesity rate is steadily increasing from 25.8% in 1988 to 35.5% in 2016 and is expected to increase rapidly by 2030 [2]. Excessive accumulation of adipose tissue in the body has been found to be a cause of various chronic diseases such as cardiovascular disease, hyperlipidemia, type 2 diabetes, high blood pressure, and chronic inflammation [3].

As the protective function against oxidative damage of natural antioxidants, such as phenolic compounds and vitamins widely present in plant foods, has been reported, interest in vegetables and wild vegetables with strong pharmacological effects has been focused [4]. Wild vegetables contain a lot of vitamins and minerals, and have antioxidant [5], antihyperlipidemic [6], and anti-obesity properties [7,8], and improvement of liver function [9]. Moreover, wild vegetables have many health benefits and functionalities to improve human life and quality, and it is evaluated that wild or cultivated wild vegetables can be used as materials for health functional foods, and medicines [4]. Butterbur (*Petasites japonicus* (Siebold & Zucc.) Maxim.) is a perennial herbaceous plant belonging to the Asteraceae family that grows naturally in wild wetlands, and it grows wild not only in China and Japan, but also in Korea, from Jeju Island to the central region [10]. In folk remedies or oriental medicine, dried sprouts were boiled in water and used for coughing, bronchial asthma, and treatment of poisoning [11]. In ‘Donguibogam’, it was recorded that *P. japonicus* (Siebold & Zucc.) Maxim. is not poisonous and that it is good to boil the stems to make soup or to eat as a side dish of herbs [12]. Among the various active ingredients present in *P. japonicus* (Siebold & Zucc.) Maxim., caffeic acid, 3-O-caffeoylquinic acid, fukinolic acid in flowers, and petasinophenol, flavonoid glycosides, phenylprophenoyl sulfonic acid, and fukinolic acid in leaves were detected as antioxidant compounds [11,12,13]. In addition, sesquiterpene esters such as petasin and s-petasin, which have anti-asthmatic and anti-allergic effects in leaves, backkenolide B, and petatewalide B with anti-allergic and anti-inflammatory effects in leaves were isolated [10]. Analysis of active components such as petasin and some phenolics from *P. japonicus* (Siebold & Zucc.) Maxim. and studies on anti-obesity effects through regulation of PPAR-γ and C/EBPα pathway signaling have been reported [14,15,16], but there is still a lack of reports related to polyphenolics analysis and in vivo efficacy for *P. japonicus* (Siebold & Zucc.) Maxim. leaves. Therefore, this study was designed to analyze the polyphenolic compounds using a HPLC and investigate the anti-obesity actions by evaluating hepatic fat and related metabolic parameters using a diet-induced obesity model.

## 2. Materials and Methods

### 2.1. Materials

Neochlorogenic acid, gallic acid, protocatechuic acid, chlorogenic acid, caffeic acid, p-hydroxybenzoic acid, 1,3-dicaffeoylquinic acid, p-coumaric acid, rutin, quercetin 3-β-galactoside, ferulic acid, taxifolin, 3,4-dicaffeoylquinic acid, trans-m-coumaric acid, quercetin 3-α-L-rhamnoside, 3,5-dicaffeoylquinic acid, 4,5-dicaffeoylquinic acid, rosmarinic acid, myrcetin, luteolin, quercetin, trans-cinnamic acid, apigenin, kaempferol, fenofibrate, dimethyl sulfoxide (DMSO), ethylenediaminetetraacetic acid (EDTA), hematoxylin, eosin, and trizma base were purchased from Sigma-Aldrich (St. Louis, MO, USA). Fetal bovine serum (FBS), Dulbecco’s modified Eagle’s medium (DMEM), streptomycin, and penicillin were purchased from Invitrogen (Carlsbad, CA, USA). Other reagents were commercially available and of special grade.

#### 2.1.1. Extraction

Fresh *P. japonicus* (Siebold & Zucc.) Maxim. was obtained from a local market in Wanju_gun, Jeollabuk-do, Republic of Korea in April 2021. The authenticity of the samples was confirmed by Professor Myung-Kon Kim from the Department of Food Science and Technology at Jeonbuk National University (voucher specimens: NIBRVP0000805454). The samples were washed with distilled water and then subjected to freeze-drying for 7 days. After that, the samples were ground into a powder using a household grinder and stored in airtight plastic bags in a freezer at −20 °C. To prepare the *P. japonicus* (Siebold & Zucc.) Maxim. extract (PJE), 5 g of frozen powder was mixed with 20 mL of 70% methanol aqueous solution and ultrasonicated at room temperature for 20 min. The mixture was then centrifuged at 4500 rpm for 10 min, and the resulting supernatant was collected. This process was repeated twice, and the collected supernatants were concentrated and vacuum evaporated at 40 °C. The resulting concentrated sample was dissolved in 10 mL of distilled water and mixed with 30 mL of saturated butanol, which was then shaken and extracted at room temperature for 2 h. The butanol layer was separated, washed with distilled water twice to remove any remaining saccharides, and then concentrated and vacuum evaporated. Finally, the concentrated sample was dissolved in 2 mL of methanol, filtered using a microsyringe filter with a pore size of 0.45 um, and used for high performance liquid chromatography (HPLC) analysis.

#### 2.1.2. HPLC Analysis

The HPLC analysis was conducted using an HPLC system with a Waters 996 DAD, and 2690 separation module, equipped with a Sunfire C18 column (5 μm, 250 mm × 4.6 mm; Waters, Milford, MA, USA). To analyze phenolic acids and flavonoids, the mobile phase consisted of 100% acetonitrile (solvent A) and 0.1% trifluoroacetic acid in deionized water (solvent B). The ratio of mobile phase was maintained at A:B = 15:85 (0–2 min), 40:60 (2–38 min), 20:80 (38–40 min), and 15:85 (40–45 min). The flow rate was 1.0 mL/min. During the HPLC analysis, the UV-Vis absorption spectra were detected between 200–400 nm, and individual compounds was analyzed by quantifying peak areas at 280 nm. The standard compounds of polyphenolics were applied to create calibration curves, including neochlorogenic acid, gallic acid, protocatechuic acid, chlorogenic acid, caffeic acid, p-hydroxybenzoic acid, 1,3-dicaffeoylquinic acid, rutin, p-coumaric acid, quercetin 3-β-galactoside, ferulic acid, taxifolin, 3,4-dicaffeoylquinic acid, trans-m-coumaric acid, quercetin 3-α-L-rhamnoside, 3,5-dicaffeoylquinic acid, 4,5-dicaffeoylquinic acid, rosmarinic acid, myrcetin, luteolin, quercetin, trans-cinnamic acid, apigenin, and kaempferol. The standard solutions (ranging from 2.9 to 1000 μg/mL) were dissolved in DMSO. The main compounds detected from PJE were identified by comparing their retention times with those of the standards. The quantities of identified compounds were determined by comparing their peak area intensities with those of the standard curves.

#### 2.1.3. Cell Culture

3T3-L1 cells were obtained from the American Type Culture Collection in Virginia, USA, and cultured in DMEM supplemented with 100 μg/mL streptomycin, 100 U/mL penicillin, and 10% FBS according to the previous method [17]. The cells were incubated in humidified air consisting of 95% air and 5% CO_2_ at 37 °C. The media were changed every 2 days. To investigate the possibility of toxic effects of PJE, 3T3-L1 cells were treated with 100–1000 μg/mL PJE for 24 h.

#### 2.1.4. Cell Viability Assay

To determine cell viability, the 3-(4,5-dimethylthiazol-2-yl)-2,5-diphenyltetrazolium bromide (MTT) reduction assay was used as described previously [17]. Cells were seeded at a density of 1 × 10^4^ cells/well into 96-well plates. After incubation for 24 h, PJE dissolved in saline solution was treated for the specified durations. After incubation of MTT (0.5 mg/mL) in each well for 4 h at 37 °C, the culture media were removed, and dimethyl sulfoxide of 100 μL was treated for 10 min in each well to dissolve the formazan crystals. Finally, the absorbance of each well was measured using a microplate reader at 570 nm, compared with wells without cells as blanks and subtracted from each sample as the background.

#### 2.1.5. Animals

Male Imprinting Control Region mice (15 weeks old and 40 to 50 g body weight ranges) were used for the diet-induced obesity (DIO) model. The mice were housed in cages and maintained under the conditions of a 12-h light/12-h dark cycle and a temperature of 22 ± 2 °C. The mice had access to either a 60% fat diet or a normal feed (LabDiet 5L79; ORIENT BIO Inc., Seongnam, Republic of Korea) and tap water ad libitum. Table 1 shows the compositions of the administered diets. To minimize animal suffering, all experimental procedures were approved by the Institutional Animal Care and Use Committee of Jeonnam Institute of Natural Resources Research, Jangheung, Republic of Korea (JINR2003), and were performed in accordance with the related ethical regulations of Gwangju University, and the National Institutes of Health Guide for the Care and Use of Laboratory Animals (NIH publication no. 80–23, revised 1996).

#### 2.1.6. In Vivo DIO Mice Model and Treatment Groups

The effect of PJE was evaluated in DIO mice. The mice were divided into 5 groups (10 mice/group) and fed a high fat diet (HFD) for 50 days. Next, 500 or 1000 mg/kg/day of PJE, and 200 mg/kg/day of fenofibrate were administrated orally for 50 days. Group 1 included normal mice treated with saline as a vehicle and fed a normal pellet diet (Control group). Group 2 included the DIO mice model treated with saline as a vehicle and fed the HFD (DIO group). Groups 3–5 comprised the DIO mice model treated with 500 mg/kg PJE (DIO + PJE500 group), 1000 mg/kg PJE (DIO + PJE1000 group), and fenofibrate (DIO + Fenofibrate group), respectively. Body weights and food intakes were recorded and the reduction (%) of body weight (BW) was calculated as 100-(final BW–initial BW)/100. The feed efficiency ratio was calculated as BW gain (final BW–initial BW)/feed mass consumed (total feed mass–remaining mass). After the administration and a 12-h fasting period, each group’s animals were anesthetized with light ether and then sacrificed, and the blood, and several tissues of liver, spleen, fats, and kidney were collected for biochemical or tissue analysis. After allowing 2 mL of clotted whole blood in a test tube, it was subjected to centrifugation at 1500× *g* for 15 min to obtain serum. The serum samples were then stored at a temperature of −70 °C to be used for further experiments. To determine the protein level, the bicinchoninic acid (BCA) assay was performed, with bovine serum albumin serving as the standard.

#### 2.1.7. Liver and Fat Tissue Histology

After an overnight fasting state, liver, and white adipose tissue (including mesenteric, epididymal, and perirenal fat) were collected and stored at −70 °C. The liver was also perfused in 10% formalin and fixed in 10% formalin for 24 h before analysis. Fat accumulation in the frozen tissue was investigated histologically using Oil Red O staining, which was analyzed for color intensity expressed as a percentage using ImageJ (NIH, Bethesda, MD, USA). The liver was observed by microscope (DM500, Leica, Heerbrugg, Switzerland). The frozen tissue was processed using a cryostat, fixed, and stained with Oil Red O.

#### 2.1.8. Biochemical Analysis

The levels of various biomarkers in serum, including total cholesterol (TC), high-density lipoprotein cholesterol (HDL), triacylglycerol (TG), glucose, and total protein (TP) were analyzed using a Vitros 250 chemistry system from Ortho Clinical Diagnostics (Ortho Clinical Diagnostics, Raritan, NJ, USA). To calculate the levels of low-density lipoprotein cholesterol (LDL) and very LDL (VLDL), the Friedewald’s formula was used [18], where LDL = TC–(HDL–TG/5) and VLDL = TG/5. The atherogenic index (AI), atherogenic coefficient (AC), cardiac risk ratio (CRR), and coronary artery index (CAI) were calculated using the equations of Ikewuchi and Ikewuchi [19]. Additionally, serum levels of adiponectin, insulin, and leptin were measured using ELISA assays (Sigma-Aldrich, St. Louis, MO, USA). The homeostasis model assessment of insulin resistance (HOMA-IR) index was calculated as (fasting serum insulin × fasting serum glucose)/22.5 [20].

#### 2.1.9. Statistical Analysis

The statistical analysis was conducted using the method as described previously [17] utilizing SPSS 21 (SPSS Inc., Chicago, IL, USA). The data obtained were presented as mean values with corresponding standard deviation (SD). To evaluate the statistical significance of comparisons between multiple groups, a one-way analysis of variance was performed, followed by a post hoc Tukey’s test. Results with *p*-values less than 0.05 were considered to be statistically significant.

## 3. Results

### 3.1. PJE Contains Polyphenolic Compounds

**Figure 1:**
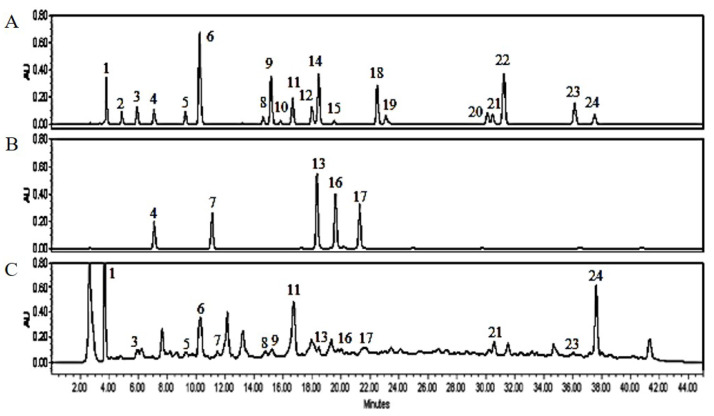
HPLC analysis in PJE. (**A**) Mixture of authentic standards, phenolic acids and flavonoids. (**B**) Mixture of authentic standards of dicaffoylquinic acids. (**C**) Polyphenolic compounds in PJE. *X*-axis is retention time in minutes and *Y*-axis is absorbance unit (AU). 1, gallic acid; 2, neochlorogenic acid; 3, protocatechuic acid; 4, chlorogenic acid; 5, p-hydroxybenzoic acid; 6, caffeic acid; 7, 1,3-dicaffeoylquinic acid; 8, rutin; 9, p-coumaric acid; 10, quercetin 3-β-galactoside; 11, ferulic acid; 12, taxifolin; 13, 3,4-dicaffeoylquinic acid; 14, trans-m-coumaric acid; 15, quercetin 3-α-L-rhamnoside; 16, 3,5-dicaffeoylquinic acid; 17, 4,5-dicaffeoylquinic acid; 18, rosmarinic acid; 19, myrcetin; 20, luteolin; 21, quercetin; 22, trans-cinnamic acid; 23, apigenin; 24, kaempferol.

We found that PJE have a large number of bio-active compounds, including flavonoids, and polyphenolics and Table 2 shows the concentrations of protocatechuic acid, gallic acid, p-hydroxybenzoic acid, chlorogenic acid, caffeic acid, 1,3-dicaffeoylquinic acid, p-coumaric acid, rutin, ferulic acid, 3,4-dicaffeoylquinic acid, 3,5-dicaffeoylquinic acid, 4,5-dicaffeoylquinic acid, quercetin, apigenin, and kaempferol detected in PJE were 34.05 ± 0.61, 4.31 ± 0.05, 20.14 ± 0.16, 60.32 ± 0.81, 15.84 ± 0.12, 0.57 ± 0.01, 9.10 ± 0.33, 2.27 ± 0.04, 34.43 ± 0.53, 18.18 ± 0.37, 4.19 ± 0.07, 3.95 ± 0.02, 15.23 ± 0.11, 1.59 ± 0.02, and 83.88 ± 1.04 μg/mL, respectively, compared to standards at each peak (Figure 1).

### 3.2. Effect of PJE on Cell Viability in 3T3-L1

**Figure 2:**
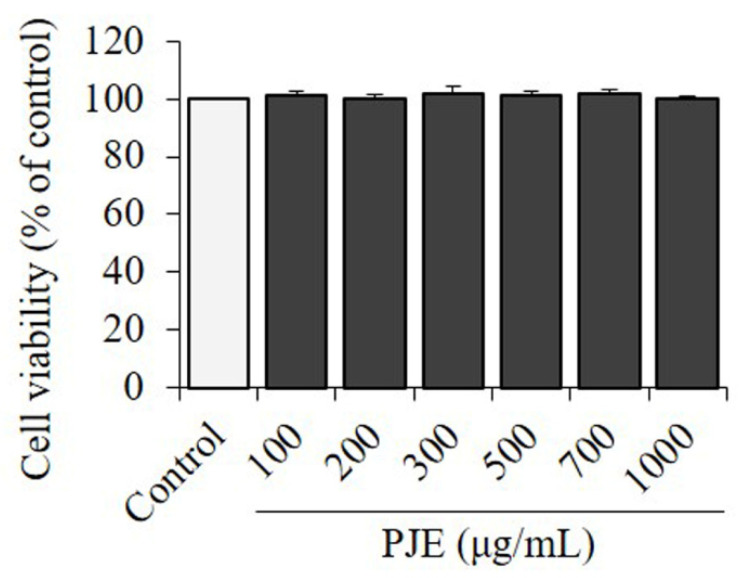
Cytotoxic effect of PJE. 3T3-L1 cells were incubated for 24 h with PJE at 0–1000 μg/mL. Cell viability was evaluated by MTT reduction assay. Each value is the mean ± SD of triplicate measurements. PJE, *Petasites japonicus* extract.

An MTT assay was conducted on the 3T3-L1 cell line to examine the effect of various concentrations (100–1000 μg/mL) of PJE on cell viability after 48 h of treatment. Although PJE treatment up to 1000 μg/mL showed no significant change on cell viability (Figure 2).

### 3.3. Effect of PJE on Body Weight Gain and Feed Efficiency of DIO Mice Model

**Figure 3:**
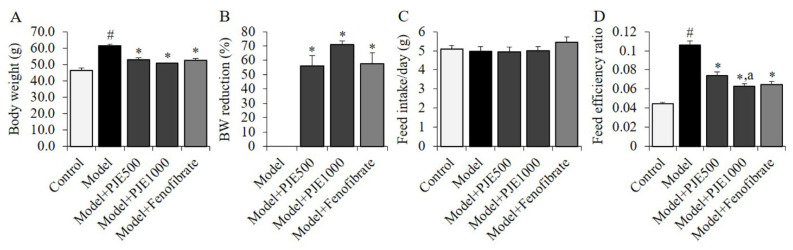
Effects of PJE and fenofibrate on body weight (**A**), reduction rate (%) of body weight (**B**), feed intake (**C**), and feed efficiency (**D**) in diet-induced obesity mice model. Each value is the mean ± SD (*n* = 10). ^#^ *p* < 0.01, compared with Control group, * *p* < 0.01, compared with DIO control group, ^a^ *p* < 0.01, compared with PJE500 group. Control, non-induced normal group; Model, diet-induced obesity model group; Model + PJE500-1000, 500–1000 mg/kg of PJE-administrated DIO group, Model + Fenofibrate, fenofibrate-administrated DIO group.

The DIO group fed with an HFD exhibited an increase in body weight compared to the control group fed with a normal diet (Figure 3A). However, the PJE and fenofibrate groups showed a significant reduction in body weight gain compared to the DIO control group (Figure 3B). There was no significant difference in mean feed intakes between the groups (Figure 3C), but the HFD resulted in an increase in feed efficiency ratio compared to the control group (Figure 3D). The administration of PJE significantly reduced the feed efficiency ratio, with a greater reduction observed in the PJE1000 group compared to the PJE500 group.

### 3.4. Effect of PJE on the Weight of Liver, Kidney, Spleen, and Fat Tissues

The effect of PJE and fenofibrate on various tissues’ weight was evaluated as shown in Table 3. The weight of the liver was increased in the DIO control group compared to the control group. However, no significant changes were observed in the weight of the spleen and kidneys. The HFD significantly increased the weights of perirenal, epididymal, and mesenteric fat tissues. On the other hand, PJE administration reduced the weight gain of fat and liver tissues, while the fenofibrate-administrated group exhibited a reduction in liver or mesenteric fat weight gain in comparison to the DIO control group.

### 3.5. Effect of PJE on Hepatic Fat in DIO Mice Model

**Figure 4:**
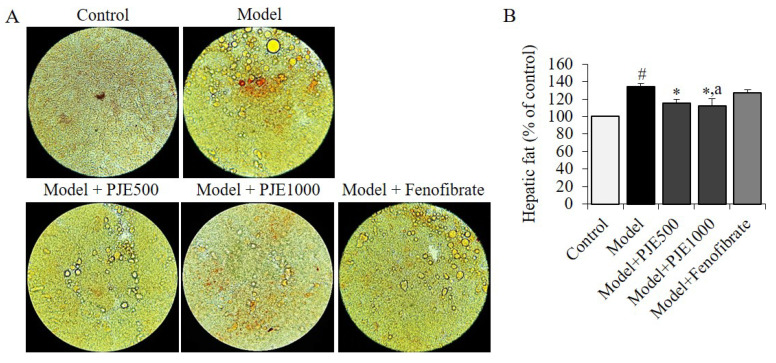
Representative histopathological analysis of the livers. Effects of PJE and fenofibrate on hepatic fat in the obesity mice model was analyzed with H&E, microscope (**A**), and ImageJ (**B**). Each value is the mean ± SD of triplicate measurements. ^#^ *p* < 0.01, compared with Control group, * *p* < 0.01, compared with DIO control group, ^a^ *p* < 0.05, compared with Fenofibrate group. Control, non-induced normal group; Model, diet-induced obesity model group; Model + PJE500-1000, 500–1000 mg/kg of PJE-administrated DIO group, Model + Fenofibrate, fenofibrate-administrated DIO group.

We conducted a study to explore how PJE or fenofibrate administration affects the accumulation of fat in the liver. Our analysis focused on the hepatic fat density in each group. The results, presented in Figure 4A,B, indicate that the consumption of a HFD caused a more rapid increase in hepatic fat accumulation than the control group. However, the administration of PJE resulted in a significant reduction in fat accumulation and density (Figure 4B) when compared to the DIO control group or the fenofibrate group.

### 3.6. Effect of PJE on Serum Biochemical Parameters

Diet-induced obesity is associated with changes in adipokines, insulin resistance, lipid profiles, cardiac risk, and atherogenesis. The levels of serum of various biochemical parameters were compared between the control group and mice fed with HFD for 50 days as shown in Table 4. The DIO control group had higher levels of most parameters, except for total protein (TP), which had an average rise; there were no significant differences in TP value. Administration with PJE resulted in lower levels of lipid parameters, including LDL, TG, TC, VLDL, and HDL compared to the DIO control group. Similarly, fenofibrate administration reduced TC, LDL, VLDL, and glucose levels. Additionally, the PJE group showed lower levels of serum insulin, insulin resistance, and glucose compared to the DIO control group. The AI, CAI, AC, and CRR of mice were increased by HFD feeding compared to the Control group (Table 4). The increased atherogenic coefficient and cardiac risk ratio indexes were markedly reduced in the PJE group, and increased levels of the coronary artery index, atherogenic coefficient, and cardiac risk ratio were strongly decreased in the fenofibrate group. Differences in parameters such as glucose, HDL, adiponectin, and total protein were not significant in the PJE500 group.

## 4. Discussion

In the present study, feeding a HFD for 50 days caused changes including increased adiposity, and liver mass, hyperlipidemia, and hepatic steatosis in the DIO model that were consistent with previous research [7,16,17,21]. Compared to mice fed a normal diet, the mice fed an HFD had a 2.32-fold increase in body weight gain, a 2.18–3.58-fold increase in body fats, and a 1.34-fold increase in hepatic fat mass and these changes might be associated with insulin resistance, hypertriglyceridemia, hypercholesterolemia, and cardiovascular-related risk factors. Additionally, the HFD led to increases in TC, TG, LDL, VLDL, glucose, and cardiovascular-related risk factors, such as AI, AC, CRR, and CAI, liver mass, insulin, and insulin resistance. The HFD also caused an increase in leptin levels and a decrease in adiponectin levels in the serum of the DIO mice model. However, administration with PJE was found to be more effective than fenofibrate as a lipid-lowering drug in reducing hepatic fat, body fat, and leptin levels. The anti-obesity effect of PJE may be due to the presence of various bio-active substances and metabolites, including kaempferol [22,23], p-hydroxybenzoic acid [24,25], ferulic acid [26,27], gallic acid [25], chlorogenic acid [27], 3,4-dicaffeoylquinic acid [28], caffeic acid [25], quercetin [22,27], rutin [27], protocatechuic acid [24,27], 3,5-dicaffeoylquinic acid [28,29], 4,5-dicaffeoylquinic acid [28,29], p-coumaric acid [25], apigenin [30,31], and 1,3-dicaffeoylquinic acid [32] in PJE, which are known for their anti-oxidative, anti-obesity, cardio-protective, and anti-inflammatory effects. Summarizing the in vivo efficacy and constituents of PJE, it contains polyphenolic substances with various physiological activities, the results of delays of the increase in body fat, cholesterol, and triglyceride, reduction in liver fat accumulation, decrease in blood glucose, insulin resistance, and leptin, and increase in adiponectin were confirmed. Utilizing these actions, it can be applied to supplements and functional foods that target fat accumulation and blood glucose reduction in obese patients that can be induced by high-fat diets.

The active components of *P. japonicus* (Siebold & Zucc.) Maxim. that are responsible for its anti-obesity effects are yet to be fully elucidated. However, several studies have reported the presence of various bioactive compounds in *P. japonicus* (Siebold & Zucc.) Maxim., such as quercetin, petasin, isopetasin, and butterbur lactones [33,34]. The flavonoid quercetin, which is abundant in many wild vegetables, has been reported to improve insulin sensitivity, reduce adipose tissue inflammation, and enhance lipid metabolism in animal models of obesity [35]. Petasins are sesquiterpene lactones and these compounds have been reported to have anti-inflammatory, anti-allergic, and analgesic properties [36]. In addition, a study by Guo et al. [37] reported that petasin inhibited adipogenesis and improved glucose uptake in 3T3-L1 adipocytes, showing that petasins are responsible for the anti-obesity efficacy of *P. japonicus* (Siebold & Zucc.) Maxim. Petasin and isopetasin, two of the main bioactive compounds in PJE, have been shown to inhibit adipocyte differentiation and adipogenesis in vitro by suppressing the expression of adipogenic transcription factors [15,36,37]. In addition, these compounds have been reported to exhibit anti-inflammatory activity by inhibiting the leukotriene synthesis in human macrophages, neutrophils, and eosinophils, and thereby suppressing the inflammatory process [38]. These findings suggest that the anti-obesity effects of PJE are mediated, at least in part, through its active compounds, petasin, isopetasin, as well as several polyphenolics.

The prevalence of obesity and its related complications such as hyperlipidemia and hypercholesterolemia have been increasing globally, leading to an urgent need for effective treatments. Obesity is a multifactorial disease that requires a multifaceted approach for treatment, and the current standard of care for its management involves lifestyle modifications, including diet and exercise, and pharmacotherapy [39]. However, the efficacy of pharmacotherapy is limited by various factors, such as adverse effects and poor patient adherence [40]. There has been growing interest in the development of novel anti-obesity drugs that target specific pathways involved in energy balance regulation [41]. Antioxidants are compounds that inhibit oxidation, a chemical reaction that produces free radicals, which are unstable molecules that can damage cells and contribute to various diseases, including obesity. The consumption of antioxidants has been linked to lower levels of oxidative stress and inflammation, both of which are involved in the development and progression of obesity [42]. Various studies have shown the potential of antioxidants in treating obesity, and some of the mechanisms involved include: inhibition of adipogenesis by quercetin and resveratrol, increase in energy expenditure by epigallocatechin gallate (EGCG), and reduction in inflammation by curcumin and anthocyanins [42,43]. Polyphenols are a group of naturally occurring compounds that are widely distributed in plants, including fruits, vegetables, and herbs, and known for their antioxidant properties, and they have been shown to have various health benefits, including anti-inflammatory, anticancer, and anti-obesity effects [44]. The metabolic processes of polyphenolics are complex and involve several mechanisms. One of the primary mechanisms by which polyphenolics exert their effects is through their interaction with gut microbiota [45]. Gut microbiota plays a critical role in the regulation of various metabolic processes, including energy homeostasis, lipid metabolism, and glucose metabolism [45,46,47]. Studies have shown that polyphenolics can modulate the composition and function of gut microbiota, which can help to improve metabolic parameters [45,48]. In addition to their effects on gut microbiota, polyphenolics can also directly modulate metabolic processes in various organs such as the liver, adipose tissue, and skeletal muscle [45,48,49], and have been found to activate AMP-activated protein kinase (AMPK), which is a key regulator of energy metabolism [50,51]. Activation of AMPK can help to increase glucose uptake and utilization, reduce lipid accumulation, and improve insulin sensitivity [51,52]. The bioavailability of polyphenolics is a crucial factor in determining their therapeutic potential. The digestive processes of polyphenolics play a crucial role in their absorption, metabolism, and bioavailability, and involve several stages [44,45,49]. The first stage is the release of polyphenolics from the plant matrix. This stage is influenced by factors such as the food matrix, and processing methods. The second stage involves the breakdown of polyphenolics by enzymes, including glycosidases and esterases. The third stage is the absorption of polyphenolics, which occurs mainly in the small intestine. Several factors can affect the absorption of polyphenolics, including their chemical structure, molecular weight, and solubility. In addition, the presence of other dietary components, such as fiber and fat, can influence the absorption of polyphenolics [53]. Once absorbed, polyphenolics are metabolized by the liver, where they undergo phase I and phase II reactions before being excreted in the urine. The digestive processes of polyphenolics have some restrictions for the potential therapeutic effects of PJE. First, the release of polyphenolics from the plant matrix is influenced by processing methods [54]. The extraction method used to obtain the PJE may impact the bioavailability of the polyphenolics. Second, the breakdown of polyphenolics by enzymes is an important determinant of their absorption. Therefore, the ability of PJE to resist enzymatic breakdown may reduce its bioavailability [55]. Third, the absorption of polyphenolics occurs mainly in the small intestine, where they compete for absorption with other dietary components. The presence of fiber or fat in the diet may reduce the absorption of polyphenolics from PJE [56]. However, the use of a standardized extract may help to ensure consistent bioavailability across different dietary conditions. The metabolism of polyphenolics by the liver can impact their therapeutic potential. The phase I and phase II reactions that occur in the liver can either enhance or reduce the bioactivity of polyphenolics. Therefore, the selection of the appropriate dose and duration of PJE administration may be critical to maximizing its therapeutic potential.

In conclusion, PJE has shown promising results in reducing obesity and related metabolic parameters, and its use as an anti-obesity drug warrants further investigation. Its potential benefits as an anti-obesity drug include its low toxicity and side effects, and its traditional use in East Asia suggests its safety and efficacy. However, further research is needed to fully understand the mechanisms underlying these effects and to determine optimal dosages and modes of administration.

## Figures and Tables

**Table 1:** Composition of the experimental diets.

Composition	Control	Model	PJE500	PJE1000	Fenofibrate
	% (*w*/*w*)				
Nitrogen-free extract	59.5	37.5	37.5	37.5	37.5
Fat	4.5	34.9	34.9	34.9	34.9
Protein	20.1	23.6	23.6	23.6	23.6
Fiber	4.6	−	−	−	−
Ash	5.8	−	−	−	−
Mineral mixture	3.5	3	3	3	3
Vitamin mixture	1	1	1	1	1
PJE500	−	−	0.5	−	−
PJE1000	−	−	−	1	−
Fenofibrate	−	−	−	−	0.2
Protein calories (%)	21.0	18.1	18.1	18.1	18.1
Fat calories (%)	13.7	61.6	61.6	61.6	61.6
Carbohydrates calories (%)	65.3	20.3	20.3	20.3	20.3
Energy (kcal/g)	4.04	4.65	4.79	4.92	4.65

Composition of the diets according to TestDiet 58Y1 and LabDiet 5L79. Model, diet-induced obesity (DIO) mice group; PJE500-1000, 500–1000 mg/kg of *Petasites japonicus* extract-administrated DIO groups; Fenofibrate, 200 mg/kg of fenofibrate-administrated DIO group. (−) components, not included.

**Table 2:** Identification and quantitation of polyphenolic compounds of PJE.

Peak no.	RT (min)	Compounds	PJE
			Concentration (μg/g, dw)
1	3.762	Gallic acid	34.05 ± 0.61
2	4.797	Neochlorogenic acid	−
3	5.849	Protocatechuic acid	4.31 ± 0.05
4	6.981	Chlorogenic acid	20.14 ± 0.16
5	9.119	p-Hydroxybenzoic acid	60.32 ± 0.81
6	10.114	Caffeic acid	15.84 ± 0.12
7	11.104	1,3-Dicaffeoylquinic acid	0.57 ± 0.01
8	14.595	Rutin	9.10 ± 0.33
9	15.049	p-Coumaric acid	2.27 ± 0.04
10	15.594	Quercetin 3-β-galactoside	−
11	16.531	Ferulic acid	34.43 ± 0.53
12	17.859	Taxifolin	−
13	18.141	3,4-Dicaffeoylquinic acid	18.18 ± 0.37
14	18.322	trans-m-Coumaricacid	−
15	19.495	Quercetin 3-α-L-rhamnoside	−
16	19.762	3,5-Dicaffeoylquinic acid	4.19 ± 0.072
17	21.381	4,5-Dicaffeoylquinic acid	3.95 ± 0.02
18	22.511	Rosmarinic acid	−
19	23.096	Myrcetin	−
20	29.969	Luteolin	−
21	30.404	Quercetin	15.23 ± 0.11
22	31.119	trans-Cinnamic acid	−
23	35.751	Apigenin	1.59 ± 0.02
24	37.437	Kaempferol	83.88 ± 1.04

The data are presented as means ± standard deviation (SD), *n* = 3. PJE, *Petasites japonicus* extract; (−) compounds, not detected or not quantified.

**Table 3:** Effect of PJE on liver, kidney, spleen, and fats tissue weights in the obesity mice model.

Parameters	Control	Model	PJE500	PJE1000	Fenofibrate
Liver	2.43 ± 0.04	3.41 ± 0.19 ^#^	2.32 ± 0.12 **	2.29 ± 0.75 **	2.10 ± 0.15 **
Kidney	0.43 ± 0.03	0.49 ± 0.05	0.45 ± 0.02	0.44 ± 0.03	0.41 ± 0.04
Spleen	0.123 ± 0.006	0.143 ± 0.009	0.129 ± 0.014	0.131 ± 0.016	0.121 ± 0.012
Epididymal fat	0.58 ± 0.09	1.85 ± 0.15 ^#^	1.35 ± 0.17 *	1.28 ± 0.25 *	1.52 ± 0.11
Perirenal fat	0.06 ± 0.01	0.20 ± 0.02 ^#^	0.16 ± 0.01 *	0.14 ± 0.02 **	0.18 ± 0.01
Mesenteric fat	0.12 ± 0.01	0.43 ± 0.04 ^#^	0.31 ± 0.01 *	0.30 ± 0.02 *	0.35 ± 0.02 **

The data are presented as means ± SD, *n* = 10. One-way ANOVA followed by the post hoc Tukey test. ^#^ *p* < 0.01, compared to Control group. * *p* < 0.05 and ** *p* < 0.01, compared to Model group. Model, diet-induced obesity (DIO) mice group; PJE500-1000, 500–1000 mg/kg of *Petasites japonicus* extract-administrated DIO groups; Fenofibrate, 200 mg/kg of fenofibrate-administrated DIO group.

**Table 4:** Effects of PJE on the levels of lipid, and associated parameters in serum from the obesity mice model.

Parameters	Control	Model	PJE500	PJE1000	Fenofibrate
TC	98.3 ± 3.5	161.0 ± 10.2 ^#^	137.2 ± 3.4 **	131.9 ± 4.1 **	110.3 ± 6.7 **
TG	85.1 ± 2.2	557.3 ± 26.3 ^#^	273.6 ± 12.8 **	255.7 ± 37.3 **	162.8 ± 5.0 **
HDL	108.0 ± 4.4	149.2 ± 1.0 ^#^	143.8 ± 5.1	147.6 ± 4.6 *	135.1 ± 6.5 **
LDL	7.3 ± 6.5	123.3 ± 10.2 ^#^	48.1 ± 7.4 **	35.4 ± 13.5 **	7.76 ± 2.5 **
VLDL	17.0 ± 0.4	111.5 ± 5.3 ^#^	54.7 ± 2.6 **	51.1 ± 7.5 **	32.6 ± 1.0 **
Glucose	204.4 ± 8.7	241.6 ± 6.1 ^#^	224.3 ± 5.9	216.1 ± 7.8 *	190.3 ± 9.2 **
Insulin	0.045 ± 0.013	0.179 ± 0.022 ^#^	0.093 ± 0.08 **	0.055 ± 0.012 **	0.037 ± 0.011 **
HOMA-IR	0.41 ± 0.02	1.92 ± 0.05 ^#^	0.93 ± 0.01 **	0.53 ± 0.03 **	0.31 ± 0.02 **
TP	5.10 ± 0.25	5.23 ± 0.33	5.25 ± 0.38	5.32 ± 0.31	5.15 ± 0.25
AI	0.79 ± 0.02	3.74 ± 0.11 ^#^	1.90 ± 0.06 **	1.73 ± 0.05 **	1.21 ± 0.04 **
AC	−0.090 ± −0.003	0.079 ± 0.002 ^#^	−0.046 ± −0.001 **	−0.106 ± −0.003 **	−0.184 ± −0.006 **
CRR	0.91 ± 0.03	1.08 ± 0.03 ^#^	0.95 ± 0.03 **	0.89 ± 0.03 **	0.82 ± 0.02 **
CAI	0.068 ± 0.002	0.826 ± 0.025 ^#^	0.335 ± 0.01 **	0.240 ± 0.007 **	0.057 ± 0.002 **
Leptin	5.06 ± 0.41	11.97 ± 1.15 ^#^	9.84 ± 0.72 *	9.11 ± 0.84 **	9.52 ± 0.59 *
Adiponectin	5.87 ± 0.13	4.95 ± 0.30 ^#^	5.07 ± 0.25	5.52 ± 0.12 *	5.59 ± 0.17 *

The data are presented as means ± SD, *n* = 10. One-way ANOVA followed by the post hoc Tukey test. ^#^ *p* < 0.01, compared to Control group. * *p* < 0.05 and ** *p* < 0.01, compared to Model group. Model, diet-induced obesity (DIO) mice group; PJE500-1000, 500–1000 mg/kg of *Petasites japonicus* extract-administrated DIO groups; Fenofibrate, 200 mg/kg of fenofibrate-administrated DIO group; TC, total cholesterol (mg/dL); TG, triacylglycerol (mg/dL); HDL, high-density lipoprotein cholesterol (mg/dL); LDL, low-density lipoprotein cholesterol (mg/dL); VLDL, very low-density lipoprotein cholesterol (mg/dL); Glucose, TP, total protein (mg/dL); Insulin (ng/mL); HOMA-IR, homeostasis model assessment-insulin resistance; AI, atherogenic index; AC, atherogenic coefficient; CRR, cardiac risk ratio; CAI, coronary artery index; Leptin (ng/mL); Adiponectin (μg/mL).

## Data Availability

Data is contained within the article.

## Sample Availability

Samples of the compounds are available from the author (Choi, J.H. and Kim, K.M.).
